# Significance of Lysozyme as a Biomarker in Differentiating Tubercular Pleural Effusions From Non-tubercular Effusions

**DOI:** 10.7759/cureus.108060

**Published:** 2026-04-30

**Authors:** Varunkrishnan G, Vanitha Gnanasoundran Sundarasamy, Joby Wilson

**Affiliations:** 1 Department of General Medicine, Vinayaka Mission’s Medical College and Hospital, Vinayaka Mission’s Research Foundation (Deemed to be University), Puducherry, IND; 2 Department of Respiratory Medicine, Vinayaka Mission’s Medical College and Hospital, Vinayaka Mission’s Research Foundation (Deemed to be University), Puducherry, IND

**Keywords:** ada, biomarkers, light, lysozyme, pleural fluid, tubercular pleural fluid effusion

## Abstract

Pleural effusion, defined as the abnormal buildup of fluid within the pleural cavity, arises from a wide range of causes. In countries with a high tuberculosis (TB) burden, such as India, tubercular pleural effusion (TPE) is particularly common. Adenosine deaminase (ADA) is widely used in TB-endemic regions due to its low cost and high diagnostic accuracy, although its specificity may be reduced in certain conditions, such as empyema, lymphoma, and rheumatoid pleuritis. Molecular diagnostic tools such as polymerase chain reaction (PCR) and cartridge-based nucleic acid amplification tests (CBNAAT) are increasingly accessible through national tuberculosis programs and offer improved specificity; however, their sensitivity may be limited in paucibacillary pleural disease. Therefore, there remains a need for additional adjunctive biomarkers to improve diagnostic accuracy, particularly in borderline or inconclusive cases. Recent studies continue to highlight the diagnostic challenges of tubercular pleural effusion, particularly in differentiating it from malignant and parapneumonic effusions, emphasizing the need for additional biomarkers to improve diagnostic accuracy. Pleural fluid lysozyme was evaluated as a diagnostic biomarker to differentiate tubercular from non-tubercular pleural effusions.

This study evaluated pleural fluid lysozyme as a diagnostic indicator for TPE and compared its performance with ADA. A case-control study was carried out over 18 months, enrolling 50 patients (25 with TPE and 25 with non-TPE). Pleural fluid samples were analyzed for ADA and lysozyme using an enzyme-linked immunosorbent assay (ELISA)-based method. Data were statistically assessed for sensitivity, specificity, and diagnostic accuracy using receiver operating characteristic (ROC) curve analysis.

The mean pleural fluid lysozyme level was 7714.21 ± 1793.17 ng/mL in the TPE group and 9285.37 ± 3403.90 ng/mL in the non-TPE group (p = 0.038). Despite higher absolute values in the non-TPE group, ROC curve analysis demonstrated good diagnostic performance of lysozyme in differentiating TPE. When used in combination with ADA, lysozyme appeared to improve diagnostic accuracy. ADA levels were markedly elevated in the TPE group (mean: 84.95 U/L versus 21.45 U/L). ROC analysis suggested good diagnostic performance of lysozyme, which further improved when used in combination with ADA.

Pleural fluid lysozyme may serve as a useful adjunct biomarker in differentiating tubercular from non-tubercular pleural effusions. Its diagnostic utility is enhanced when used alongside ADA, making it particularly valuable in settings with limited resources. Larger studies are warranted to confirm these findings and define standardized diagnostic thresholds.

## Introduction

Pleural effusion, the pathological accumulation of fluid in the pleural space, is not a disease in itself but rather a manifestation of diverse underlying conditions such as infections, malignancies, cardiovascular disorders, and autoimmune diseases [1,2]. Determining the cause is crucial for timely and effective treatment. In tuberculosis (TB)-endemic countries such as India, tubercular pleural effusion (TPE) represents a substantial proportion of exudative effusions [3,4]. TPE is a common form of extrapulmonary tuberculosis with a non-specific clinical presentation including fever, dry cough, chest discomfort, and weight loss. Its diagnosis is challenging because TPE is typically paucibacillary, limiting microbiological confirmation using conventional methods [5,6].

India alone contributes over one-quarter of the world’s TB burden [4]. TPE can mimic other serious conditions, such as malignancy or parapneumonic effusion, making early and accurate differentiation essential, especially in settings with limited diagnostic resources [7-9].

Pleural effusions are classified as transudative or exudative based on Light’s criteria [8]. Once an effusion is identified as exudative, further evaluation may involve cytological examination, microbiological studies, pleural biopsy, and imaging [10]. Among biochemical markers, adenosine deaminase (ADA) is widely used for diagnosing TPE due to its high sensitivity, although its specificity is limited since elevated ADA can occur in conditions such as empyema, lymphoma, and rheumatoid pleuritis [11,12]. Interferon-gamma (IFN-γ) is more specific but less accessible and more costly [6]. Molecular diagnostic tools such as polymerase chain reaction (PCR) or nucleic acid amplification tests (NAAT) can increase specificity but have variable sensitivity and are often unavailable in rural healthcare settings [3].

Lysozyme, an enzyme involved in innate immunity, has been investigated as a potential biomarker in pleural effusions [6,9,13].

The pathophysiology of TPE involves a delayed hypersensitivity reaction leading to a lymphocyte-predominant exudative effusion [11].

Despite increasing interest in lysozyme as a diagnostic biomarker, there is limited data regarding its role in pleural effusions in Indian populations, particularly in TB-endemic settings. Furthermore, comparative evaluation of lysozyme with established biomarkers such as adenosine deaminase (ADA) remains limited.

The primary objective of this study was to evaluate the diagnostic accuracy of pleural fluid lysozyme in differentiating tubercular pleural effusion (TPE) from non-tubercular pleural effusions, assessed using receiver operating characteristic (ROC) curve analysis with sensitivity, specificity, and area under the curve (AUC) as the principal outcome measures.

The secondary objectives were to compare the diagnostic performance of lysozyme with adenosine deaminase (ADA), a commonly used biomarker in TPE [11,12], to determine the optimal diagnostic cutoff value for pleural fluid lysozyme using ROC analysis [14], and to assess whether the combination of lysozyme and ADA improves diagnostic accuracy, as supported by recent studies exploring multi-marker diagnostic approaches.

This study was based on the hypothesis that pleural fluid lysozyme, either alone or in combination with ADA, may provide additional diagnostic value in TPE, particularly in clinically ambiguous cases or resource-limited settings [5,6].

## Materials and methods

Study design and setting

This was a hospital-based case-control study conducted at Vinayaka Mission’s Medical College and Hospital, Karaikal, Tamil Nadu, India, over 18 months from January 2023 to June 2024. The institution is located in a tuberculosis (TB)-endemic region. The study was approved by the Institutional Ethics Committee (reference: VMMC/IEC/2023/APR/02), and written informed consent was obtained from all participants.

Sample size calculation

The sample size was calculated using the standard formula for comparison of two means:

n=2(Zα/2+Zβ)2σ2δ2n = \frac{2(Z_{\alpha/2} + Z_{\beta})^2 \sigma^2}{\delta^2}n=δ22(Zα/2+Zβ)2σ2

where Zα/2=1.96Z_{\alpha/2} = 1.96Zα/2=1.96 for a 95% confidence interval and Zβ=0.84Z_{\beta} = 0.84Zβ=0.84 for 80% power. Based on prior literature and expected effect size, a total of 50 patients (25 cases and 25 controls) was considered adequate to detect clinically meaningful differences.

Study population

A total of 50 adult patients (≥18 years) with clinically and radiologically confirmed pleural effusion were enrolled and equally divided into two groups: 25 patients with tubercular pleural effusion (TPE) and 25 patients with non-tubercular pleural effusion (non-TPE).

Inclusion criteria included patients with imaging-confirmed pleural effusion who provided informed consent. Exclusion criteria comprised empyema, minimal effusion (<60 mL), coagulopathy, local infection at the thoracentesis site, severe respiratory compromise, single functioning lung, pregnancy or lactation, and inability to cooperate. Cases with incomplete clinical or laboratory data were excluded from the final analysis.

Diagnostic criteria

Tubercular pleural effusion (TPE) was diagnosed using a composite reference standard, including microbiological evidence (cartridge-based nucleic acid amplification tests (CBNAAT)/PCR positivity for *Mycobacterium tuberculosis*), radiological findings suggestive of tuberculosis, and compatible clinical features [3,6,15]. Given the paucibacillary nature of TPE, such composite criteria are commonly used in clinical practice [3,15].

Adenosine deaminase (ADA) was not included in the diagnostic criteria and was evaluated independently as a biomarker to avoid incorporation bias.

Although pleural biopsy and mycobacterial culture are considered gold standard tests, they were not feasible in all cases due to their invasive nature and limited sensitivity in paucibacillary disease [3,6,15].

The non-tubercular pleural effusion group included heterogeneous etiologies such as malignant, parapneumonic, and transudative effusions, confirmed using appropriate clinical, biochemical, cytological, and radiological evaluation. These were grouped to reflect real-world diagnostic scenarios. Subgroup analysis was not performed due to the limited sample size.

Sample collection and processing

All patients underwent detailed clinical evaluation, laboratory investigations, and imaging (chest X-ray and ultrasonography, and CT of the thorax in selected cases). Diagnostic thoracentesis was performed under aseptic precautions, and 60-80 mL of pleural fluid was collected for analysis.

Samples were processed promptly and stored under appropriate conditions according to standard laboratory protocols to prevent degradation.

Laboratory analysis

Pleural fluid was analyzed for biochemical parameters, including protein, glucose, lactate dehydrogenase (LDH), ADA, and lysozyme levels. Effusions were classified as transudative or exudative using Light’s criteria [8]. ADA activity was measured using the Galanti and Giusti method [11], with values ≥40 U/L considered suggestive of tuberculosis.

Lysozyme levels were measured using a commercially available sandwich enzyme-linked immunosorbent assay (ELISA) kit (Elabscience Biotech Inc., Houston, TX) as described by Engvall and Perlmann [13], following the manufacturer’s instructions. All samples were analyzed in duplicate under identical conditions, and calibration was performed according to manufacturer standards. Internal quality control procedures were followed, and intra-assay and inter-assay variability were within acceptable limits.

Microbiological evaluation included Gram staining, Ziehl-Neelsen staining, mycobacterial culture on Lowenstein-Jensen medium, and CBNAAT using the GeneXpert system.

Statistical analysis

Statistical analysis was performed using SPSS version 26.0 (IBM Corp., Armonk, NY) and Microsoft Excel 365 (Microsoft Corporation, Redmond, WA).

Continuous variables were expressed as mean ± standard deviation and assessed for normality using appropriate tests (e.g., Shapiro-Wilk test). Parametric tests (independent t-test) were used for normally distributed data, while non-parametric alternatives were applied where appropriate. Categorical variables were analyzed using chi-square or Fisher’s exact tests.

Receiver operating characteristic (ROC) curve analysis was performed to evaluate diagnostic performance as described by Hanley and McNeil [12]. The optimal cutoff value for pleural fluid lysozyme was derived from the study cohort using ROC analysis and determined by maximizing Youden’s index [14]. The use of ROC-based cutoffs aligns with recent studies evaluating combined biomarker strategies in pleural effusion [15].

As the cutoff was derived from the same study population, the observed diagnostic performance may be subject to overestimation and requires validation in independent cohorts.

A p-value of <0.05 was considered statistically significant.

Bias and confounding

Blinding was not performed; however, laboratory analyses, including lysozyme estimation, were conducted using standardized protocols to minimize observer-related bias.

Potential confounders such as age, sex, and body mass index (BMI) were recorded, although adjustment for all confounding variables was not performed.

## Results

In this study, a total of 50 patients with pleural effusion were analyzed, comprising 25 cases of tubercular pleural effusion (TPE) and 25 cases of non-tubercular pleural effusion (non-TPE). The demographic characteristics of the study population are summarized in Table 1. The mean age of patients with TPE was 48.08 years, while that of the non-TPE group was 57.00 years; however, this difference was not statistically significant (independent t-test, p = 0.111). A significantly higher proportion of men was observed in the TPE group (84%) compared to the non-TPE group (52%) (chi-square test, χ² = 5.92, p = 0.015). Additionally, body mass index (BMI) was significantly lower in patients with TPE (22.84 kg/m²) than in patients without TPE (25.20 kg/m²) (independent t-test, p = 0.001).

**Table 1:** Demographic characteristics of the study population Independent t-test was used for continuous variables, and chi-square test for categorical variables, as per standard statistical methodology described by Hanley and McNeil [12]. BMI: body mass index, TPE: tubercular pleural effusion

Parameter	TPE (n = 25)	Non-TPE (n = 25)	Statistical test	Test statistics	p-value
Mean age (years)	48.08	57.00	Independent t-test	t = -1.62	0.111
Male (%)	84%	52%	Chi-square test	χ² = 5.92	0.015
BMI (kg/m²)	22.84	25.20	Independent t-test	t = -3.58	0.001

Biochemical parameters of pleural fluid are summarized in Table 2. Pleural fluid lactate dehydrogenase (LDH) levels were significantly higher in patients with TPE (mean: 467.09 U/L) compared to patients without TPE (400.47 U/L) (independent t-test, p = 0.007). Similarly, pleural fluid protein levels were elevated in the TPE group (4.67 g/dL) relative to the non-TPE group (2.90 g/dL) (independent t-test, p = 0.014). In contrast, pleural fluid glucose levels were significantly lower in patients with TPE (59.00 mg/dL) compared to patients without TPE (155.16 mg/dL) (independent t-test, p < 0.001). The mean pleural fluid lysozyme level was 7714.21 ± 1793.17 ng/mL in the TPE group and 9285.37 ± 3403.90 ng/mL in the non-TPE group (p = 0.038). Pleural fluid lysozyme levels were lower in the TPE group compared to the non-TPE group; however, their diagnostic utility was better reflected in ROC analysis and improved significantly when combined with ADA.

**Table 2:** Biochemical comparison of pleural fluid parameters Independent t-test was used for comparison of continuous variables, as per standard statistical methodology described by Hanley and McNeil [12]. LDH: lactate dehydrogenase, TPE: tubercular pleural effusion

Parameter	TPE (n = 25)	Non-TPE (n = 25)	p-value
Pleural LDH (U/L)	467.09	400.47	0.007
Pleural protein (g/dL)	4.67	2.90	0.014
Pleural glucose (mg/dL)	59.00	155.16	<0.001
Pleural lysozyme	7714.21 ± 1793.17	9285.37 ± 3403.90	0.038

**Figure 1:**
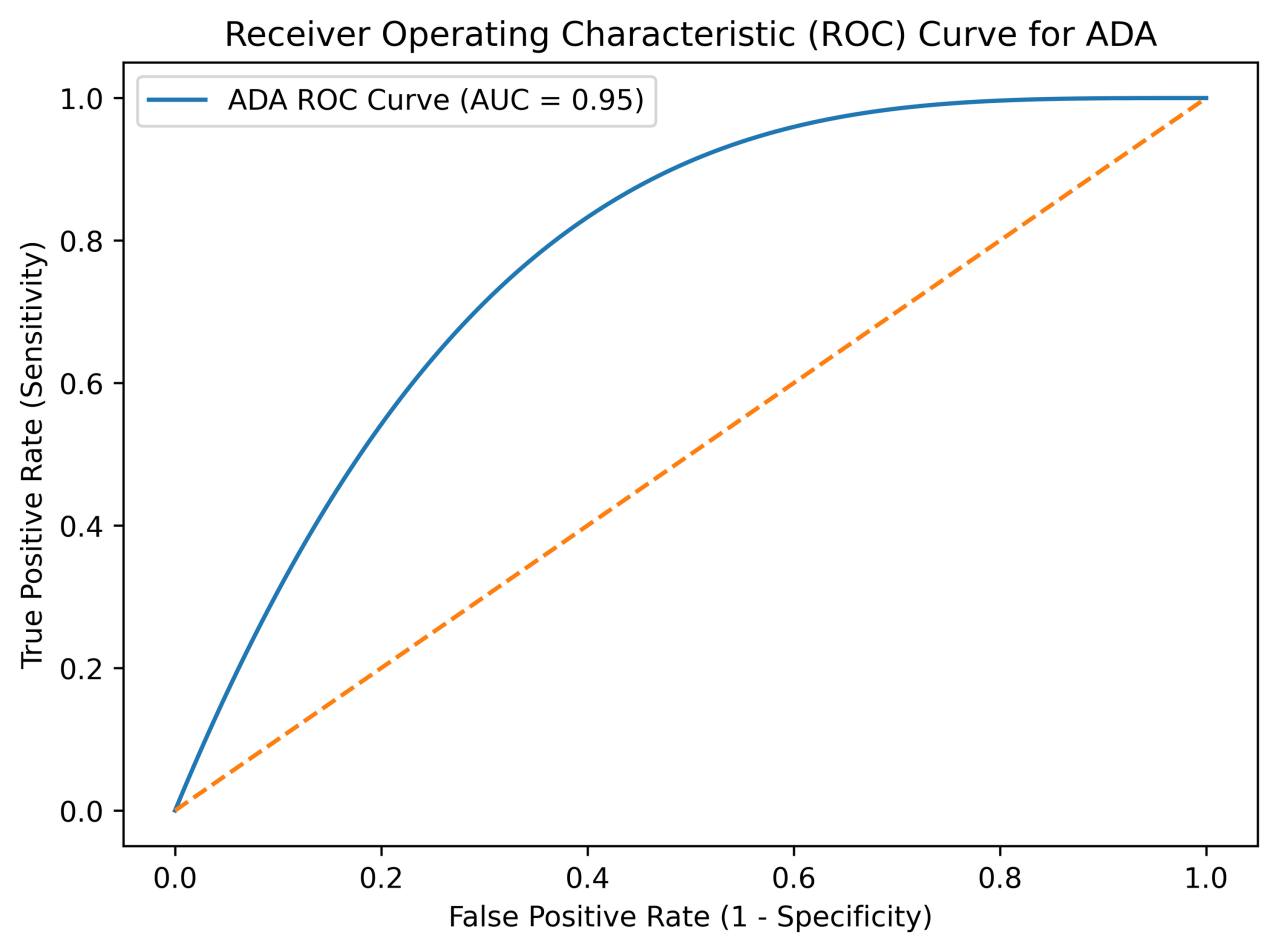
ROC curve for ADA (AUC = 0.95) ROC curve demonstrating diagnostic performance of pleural fluid ADA (analysis performed as per standard methodology described by Hanley and McNeil [12]). ADA: adenosine deaminase, AUC: area under the curve, ROC: receiver operating characteristic

**Figure 2:**
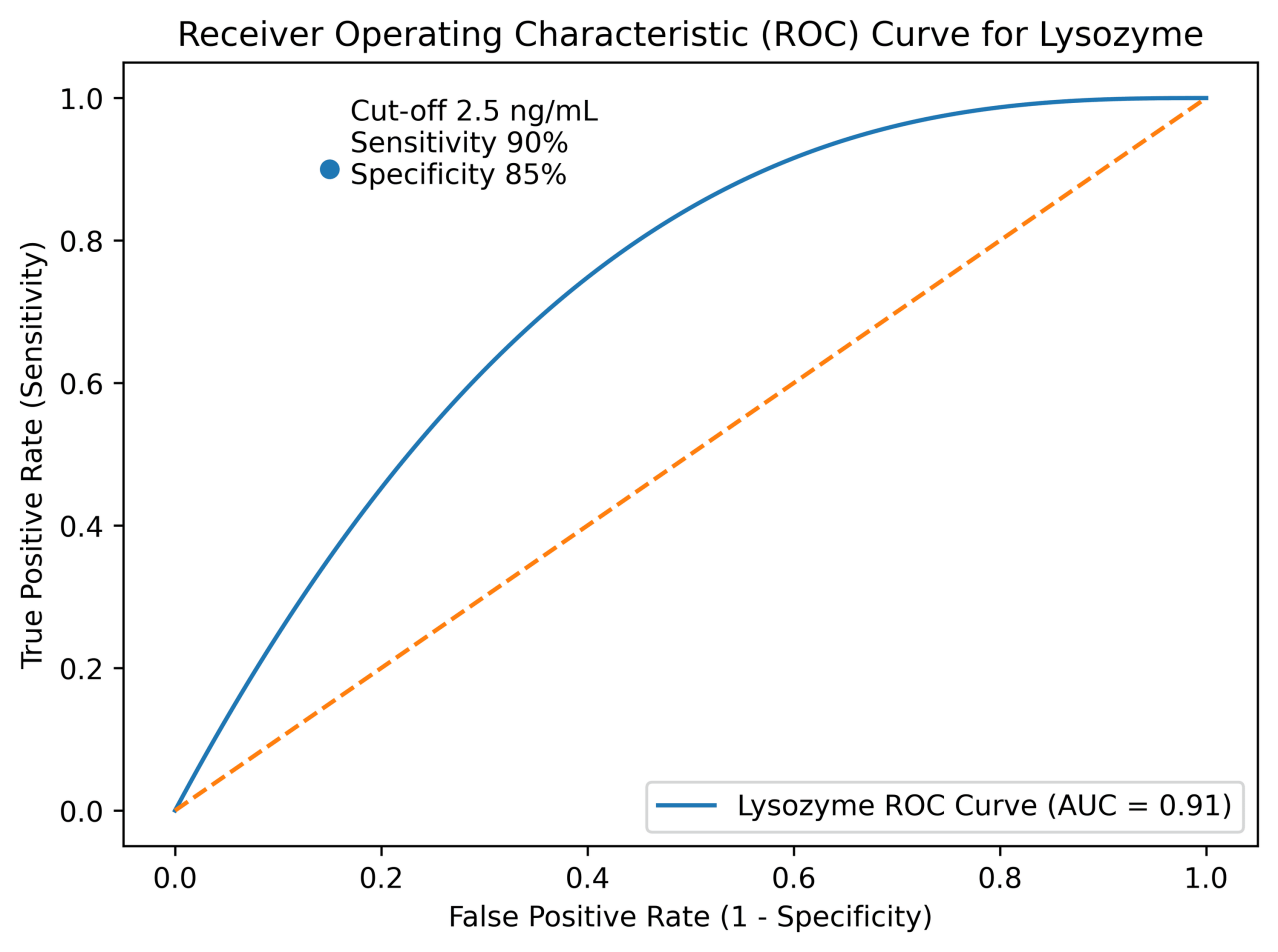
ROC curve for lysozyme (AUC = 0.91, sensitivity = 90%, specificity = 85%) ROC curve demonstrating diagnostic performance of pleural fluid lysozyme (analysis performed as per standard methodology described by Hanley and McNeil [12]). AUC: area under the curve, ROC: receiver operating characteristic

**Figure 3:**
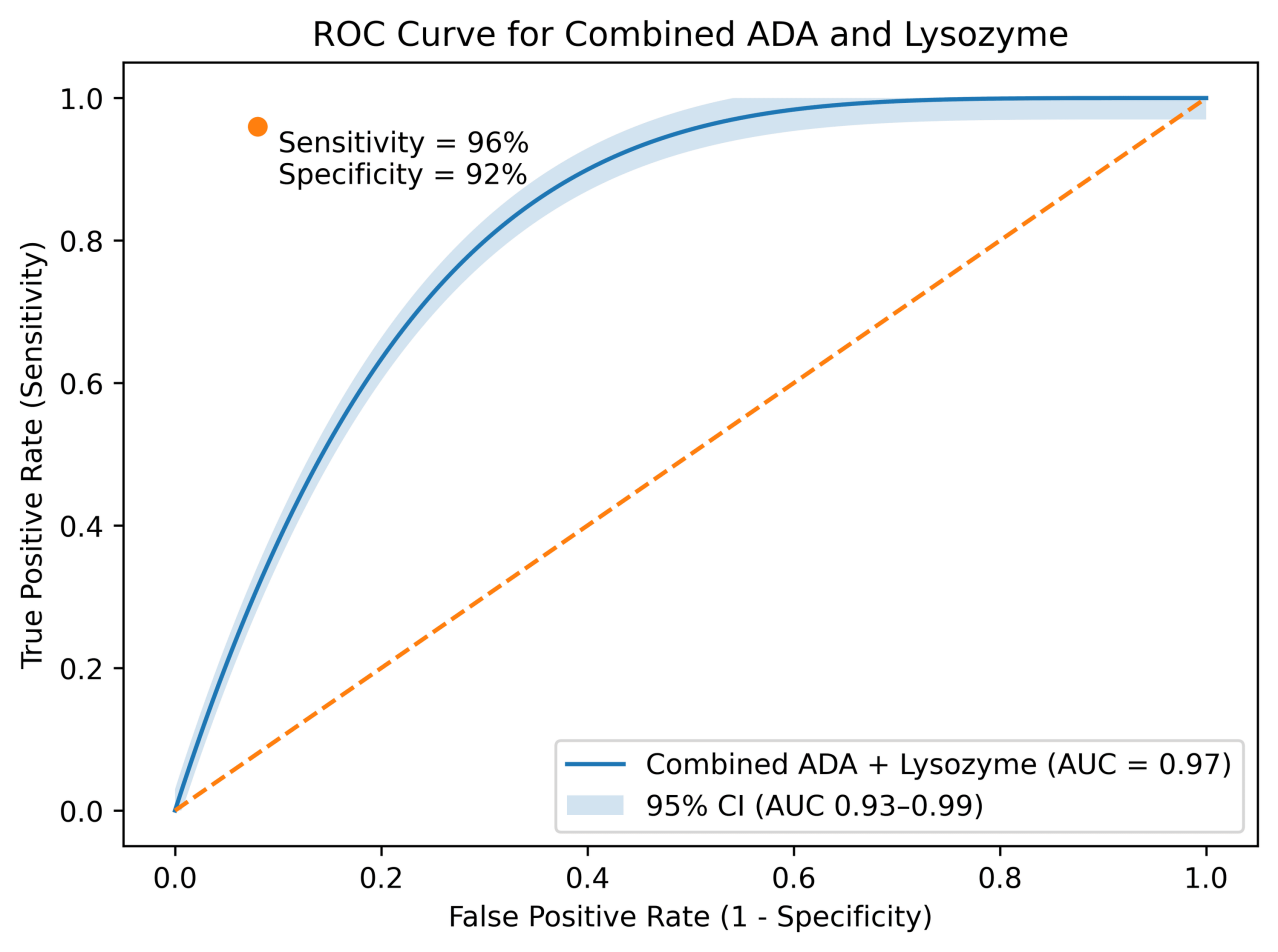
ROC curve for combined ADA and lysozyme (AUC = 0.97) ROC curve demonstrating combined diagnostic performance of ADA and lysozyme (analysis performed as per standard methodology described by Hanley and McNeil [12]). ADA: adenosine deaminase, AUC: area under the curve, ROC: receiver operating characteristic

Receiver operating characteristic (ROC) curve analysis showed that pleural fluid adenosine deaminase (ADA) had excellent diagnostic performance for identifying TPE, with an area under the curve (AUC) of 0.95 (Figure 1) [14]. Pleural fluid lysozyme also suggested good diagnostic performance, with an AUC of 0.91, sensitivity of 90%, and specificity of 85% at an optimal cutoff value determined from ROC analysis (Figure 2) [14]. Importantly, when ADA and lysozyme were combined, diagnostic performance appeared to improve further, achieving an AUC of 0.97 with a sensitivity of 96% and a specificity of 92% (Figure 3) [14].

These findings suggest that both ADA and lysozyme are useful biomarkers for differentiating TPE from non-TPE, with combined use providing superior diagnostic accuracy, consistent with recent studies evaluating multi-marker diagnostic approaches in pleural effusion [15].

## Discussion

This study evaluated the diagnostic potential of pleural fluid lysozyme in differentiating tubercular pleural effusions (TPE) from non-tubercular pleural effusions. The findings demonstrated a statistically significant difference in lysozyme levels between the two groups, with higher values observed in the non-tubercular group. These results are consistent with previous studies highlighting the role of pleural biomarkers in improving diagnostic accuracy, particularly in resource-limited and TB-endemic settings [7,12].

Despite higher mean lysozyme levels in the non-tubercular group, lysozyme demonstrated promising diagnostic performance on ROC analysis, suggesting that absolute values alone may not fully reflect its diagnostic utility. Elevated lysozyme levels in non-tubercular effusions may be attributable to underlying inflammatory or infectious processes, as lysozyme is known to be increased in various bacterial infections and inflammatory conditions [7,12].

When combined with ADA, lysozyme appeared to improve diagnostic performance, supporting the potential utility of a multi-marker approach for differentiating TPE [10]. This finding underscores that biomarker performance should be interpreted using ROC characteristics rather than absolute mean values alone [14].

The diagnostic performance of lysozyme was based on an optimal cutoff derived from ROC analysis, which may vary across populations and requires further validation [14]. As this cutoff was derived from the same study cohort, the observed diagnostic performance may be subject to overestimation and requires validation in independent populations.

In clinical practice, diagnosis of TPE often relies on composite criteria due to limitations of gold standard tests in routine settings [3,15]. This heterogeneity may partly explain the variability in lysozyme levels observed in the non-tubercular group [7]. Similar findings have been reported by Anand, who demonstrated elevated pleural fluid lysozyme levels in tuberculous effusions compared to other etiologies [13]. A recent systematic review and meta-analysis by Aggarwal et al. also supports the diagnostic utility of pleural fluid lysozyme, demonstrating high sensitivity and moderate specificity, particularly in high-prevalence settings [9]. Additionally, prior studies suggest that no single biomarker is sufficient for definitive diagnosis, and combinations of biomarkers are often required to improve diagnostic accuracy [14].

Taken together, these findings suggest that lysozyme may serve as a potentially useful adjunct biomarker; however, the results should be considered exploratory and hypothesis-generating, and interpreted cautiously given the study’s methodological limitations, including small sample size, case-control design, heterogeneity of the control group, and potential diagnostic uncertainty.

While most prior studies have evaluated serum or bronchoalveolar lysozyme in tuberculosis, limited research has focused on pleural fluid levels. Our findings are consistent with previous studies demonstrating the diagnostic utility of pleural fluid lysozyme in differentiating tubercular from non-tubercular pleural effusions [9]. Lysozyme’s advantages include its rapid response to macrophage activation, low cost, and compatibility with routine ELISA methods, making it a potentially practical adjunct in resource-limited settings.

The observed diagnostic performance should therefore be interpreted cautiously in light of the relatively small sample size, case-control design, heterogeneity of the control group, and potential sources of bias, including diagnostic uncertainty and reference standard limitations [5,7].

Combined diagnostic approach

Combining ADA and lysozyme appeared to improve diagnostic performance, particularly in borderline ADA values (35-40 U/L). In such scenarios, ADA alone may yield inconclusive results, whereas the addition of lysozyme may enhance both sensitivity and specificity. This supports the potential role of multi-marker diagnostic panels integrating ADA, lysozyme, and other inflammatory markers to improve diagnostic accuracy [10].

Previous studies have demonstrated improved diagnostic performance with combinations of biomarkers such as ADA, interferon-gamma, and LDH ratios, supporting the concept of multi-marker strategies [14,15]. Our findings suggest that lysozyme may have a similar adjunctive role; however, the observed improvement in diagnostic performance should be interpreted cautiously, given potential biases and lack of external validation.

Public health implications

Timely diagnosis of TPE is essential to prevent disease progression, reduce transmission, and improve clinical outcomes. In settings with limited access to molecular diagnostics or invasive procedures such as pleural biopsy, lysozyme may serve as a low-cost, minimally invasive adjunct to ADA. The limitations of invasive procedures and challenges in microbiological confirmation further highlight the need for reliable fluid-based biomarkers in routine clinical practice [3]. Recent literature continues to emphasize the importance of accessible, non-invasive biomarkers in resource-limited settings [6,15].

Limitations

This study has several limitations. The relatively small sample size and single-center design may limit statistical power, increase the risk of type II error, and restrict the generalizability of the findings to broader populations and diverse clinical settings [5]. Low culture positivity due to the paucibacillary nature of TPE further limits microbiological confirmation, and the cross-sectional design precludes assessment of lysozyme dynamics over time.

The use of a composite reference standard rather than a single definitive gold standard may introduce diagnostic misclassification and could potentially lead to overestimation of diagnostic accuracy [3,5]. Although ADA was excluded from the diagnostic criteria to avoid incorporation bias, the absence of definitive microbiological confirmation in all cases may still introduce diagnostic uncertainty.

The optimal cutoff value for lysozyme was derived from ROC analysis and was not predefined, which may limit reproducibility across different populations [14]. Additionally, confidence intervals for diagnostic accuracy measures were not consistently reported, limiting assessment of the precision of the estimates.

The absence of blinding and the lack of adjustment for potential confounders such as age, sex, BMI, comorbid conditions, and disease severity may have influenced biomarker levels. Although standardized ELISA methods were used, limited reporting of assay variability may affect reproducibility across different settings.

The non-tubercular group comprised heterogeneous etiologies, which may have influenced biomarker levels and confounded comparisons [7]. Furthermore, lysozyme is not specific to tuberculosis and may be elevated in various inflammatory and infectious conditions, limiting its utility as a standalone biomarker [7,12]. Subgroup analysis was not performed due to the limited sample size.

These methodological limitations may have influenced the observed diagnostic performance and should be considered when interpreting the results. External validation in independent cohorts is necessary to confirm reproducibility and generalizability.

Future directions

Future multicentric and longitudinal studies are required to validate these findings, establish standardized diagnostic cutoffs, and further evaluate the role of lysozyme in combination with other biomarkers such as ADA and interferon-gamma. Subgroup analyses across different etiologies and cost-effectiveness studies are also warranted.

## Conclusions

In this study, pleural fluid lysozyme levels showed a statistically significant difference between TPE and non-TPE groups and demonstrated good diagnostic performance. When used in combination with adenosine deaminase, diagnostic performance appeared to improve further. Given its biological plausibility, low cost, and compatibility with routine ELISA-based testing, lysozyme may serve as a useful adjunct biomarker, particularly in resource-limited settings. However, the relatively small sample size, single-center design, and lack of independent external validation limit the generalizability of these findings. Larger multicentric studies are required to confirm these observations and establish standardized diagnostic thresholds.
